# Data needs for accelerating research at the intersection of climate stressors and health: an online survey

**DOI:** 10.1088/2752-5309/ae44c0

**Published:** 2026-02-24

**Authors:** Emma L Gause, Keith R Spangler, Heather Clifford, Michaela Hoenig, Joshua S Cetron, Zachary Popp, Michelle Audirac, Julie Goldman, Amruta Nori-Sarma, Francesca Dominici, Gregory A Wellenius, Danielle Braun, Kevin Lane

**Affiliations:** 1 Center for Climate and Health, Boston University School of Public Health, Boston, MA, United States of America; 2 Department of Health Sciences, Boston University Sargent College of Health & Rehabilitation Sciences, Boston, MA, United States of America; 3 Institute for Global Sustainability, Boston University, Boston, MA, United States of America; 4 Department of Epidemiology, Boston University School of Public Health, Boston, MA, United States of America; 5 Institute for Quantitative Social Science, Harvard University, Cambridge, MA, United States of America; 6 Department of Environmental Health, Boston University School of Public Health, Boston, MA, United States of America; 7 Department of Biostatistics, Harvard T.H. Chan School of Public Health, Boston, MA, United States of America; 8 Countway Library of Medicine, Harvard Medical School, Boston, MA, United States of America; 9 Department of Environmental Health, Harvard T.H. Chan School of Public Health, Cambridge, MA, United States of America; 10 Department of Data Science, Dana-Farber Cancer Institute, Boston, MA, United States of America

**Keywords:** data needs, climate and health, research infrastructure

## Abstract

Assessing the health impacts of climate stressors is challenging due to the inherently interdisciplinary nature of the effort and the complexity of data, methods, and software involved. We surveyed researchers who published in the climate and health space to identify major barriers to using and sharing climate and health data and code resources. Participants were identified using a PubMed query to return articles related to research in the field of climate and health. Using the PubMed API, we scraped email addresses for authors of matching published articles. 9195 authors were emailed a link to the online survey instrument, which took approximately 7 min to complete. We had an 11.8% response rate resulting in 1041 useable responses. Respondents were from over 75 different countries with only 16.4% working with US populations and were evenly represented between early, mid, and established career. The most desired resources were analysis-ready datasets and educational materials on data management and analysis. Personal constraints such as lack of time were a major barrier to sharing data or code. Our survey results suggest that investment in data creation as a professional service, knowledge sharing and collaboration, and research infrastructure will be enthusiastically adopted and help to accelerate the pace of research to practice.

## 1. Introduction

The world’s climate has an immense impact on human health and wellbeing. Climate-related hazards such as extreme heat, extreme precipitation and weather events, drought, and wildfire all have direct effects on individual and population health. Climate hazards can also exacerbate existing health-related vulnerabilities, such as by altering environmental suitability for infectious disease vectors, contributing to food and drinking water insecurity, spurring economic shocks, and increasing other health stressors [1]. The World Health Organization (WHO) estimates that between 2030 and 2050, climate-related stressors will result in an excess of 250 000 deaths per year from undernutrition, malaria, diarrhea, and heat stress, among other impacts [2]. Scholars estimate that deaths resulting from climate hazards exceed the combined death toll from every major WHO-recognized public health emergency [3], emphasizing the need for further investment into disrupting harmful associations between these hazards and human health.

Researching the health impacts of climate-related stressors is challenging due to the inherently interdisciplinary nature of the effort and the complexity of data, methods, and software involved. Successful collaboration requires communication across the often siloed disciplines of earth science, public health, data science, and countless others, all of whom have their own lexicons for addressing similar concepts. Curating a robust and accessible research infrastructure that can be easily navigated across disciplines will help promote collaboration and accelerate the pace of climate-stressor and health research. However, it is critical to first ascertain the most salient barriers identified by researchers engaged in this work to prioritize investment into addressing the identified gaps. A survey of National Science Foundation (NSF) funded principal investigators in 2013 identified that almost all respondents would be engaging with large datasets and thus required additional support in the form of data management and sharing capacity, computational power, replicable workflows, and bioinformatics help in order to be successful [4]; these needs are still common and growing today. Another study of climate migration researchers and practitioners identified the need for clarified and shared understanding of common definitions as well as better available data to close research gaps and therefore influence policy [5]. A focus on cultivating these shared data and infrastructure resources will ultimately make it easier for researchers to proceed to the science.

With this in mind, we conducted a large-scale survey of researchers who have published in the climate and health space in an effort to gain insights into the technical data and software needs of this community of practice. The main goals were to identify commonly used data elements, understand potential barriers or facilitators for using and sharing data, and similar concepts for code and analysis workflows.

## 2. Methods

### 2.1. Study population

Our target survey population included individuals working at the intersection of climate and health or adjacent fields with an interest in moving into this space. To identify these individuals, we conducted a search within PubMed looking for authors of articles written in English related to climate stressors and their health consequences published in 2021 through 2023. Climate stressors include any climate hazard that may impact human health, such as extreme temperatures, extreme precipitation or drought, other extreme weather including storms, climate-related air pollution, changes in vector ecology due to climate, food or water insecurity as a result of climatic patterns, etc. The specific PubMed query is described in detail in the supplementary materials (eMethods 1). Email addresses for the authors of the identified climate and health articles were scraped using the easyPubMed [6] R package (RRID:SCR_023570) that queries the National Center for Biotechnology Information PubMed API. Retrieved author emails were most frequently those of the corresponding author, but some publications listed emails for all co-authors, and many publications had no email address recorded in the system. Efforts were made to exclude duplicate contacts by comparing unique email addresses with last names and first initials of listed authors.

### 2.2. Survey dissemination

Survey questions related to the data and software needs of the climate and health community were developed internally by the study team with several rounds of internal edits and review, and finally by pilot testing within the internal team (eMethods 2) [7]. Survey questions were developed to inform the activities of the study team with plans to share the results broadly to promote transparency and to help guide the field towards the most desired and necessary initiatives. The survey questionnaire was programmed into Research Electronic Data Capture (REDCap) [8] (RRID:SCR_003445) for dissemination. REDCap is a secure web-based data capture tool hosted at Boston University (CTSI 1UL1TR001430) which allows for validated questionnaires, automated distribution and reminders via email, participant response tracking, and seamless data downloads for subsequent analysis of the results using common statistical packages. The survey was designed to take less than 10 min to complete and this timing was verified by internal survey testers prior to deployment. The survey was administered online in English only. Participants were informed that their response to the survey was considered their consent to the study.

Initial survey invitations were emailed to participants on Monday, 18 March 2024. Participants who had not yet responded to the survey were emailed a reminder every 8 d for three reminders and a total of four contacts. Each reminder was scheduled to arrive on a different day of the week to accommodate respondents’ diverse schedules. Due to an issue with the automatic notification alerts, the first reminder email was sent to participants a few days early. The survey was closed on 3 May 2024, at which point no new responses were accepted.

All invited survey participants were asked two questions to determine their eligibility within our target population. Participants were asked whether they currently ‘engaged in research or practice related to climate and health’ and, if not, whether they were ‘interested in working in the climate and health space, collaborating with health scientists, and/or contributing data to the climate/health research field.’ If participants responded ‘no’ to both screening questions, a stop action was triggered letting the participant know they were not the intended audience.

### 2.3. Calculating survey response rates

Response rates were calculated using the American Association for Public Opinion Research standard definitions for an internet survey with specifically named persons [9]. We included both complete and partial survey returns as responses and adjusted the response rate by estimating the proportion of non-responders that were not eligible using the percent eligible among those who did respond (i.e. respondents who triggered a stop action on the survey because they were not within our intended target population). This estimation is likely an under-estimate of true eligibility as it relies on surveys that were returned to us while it is much more likely that non-eligible individuals might simply choose not to respond.

### 2.4. Response analysis

Survey responses were assessed overall as well as stratified by career duration, and a United States (US) vs. non-US research focus. Career duration strata were defined using the survey question, ‘How long have you been working in the climate and health field?’ and were categorized into three groups corresponding to early career (less than 5 years), mid-career (5–10 years), and established career (more than 10 years). US-focused respondents were identified using the yes/no question, ‘Is your research or practice primarily focused on US populations?’ Descriptive statistics were used to analyze all categorical questions. Results of the career duration stratification are presented here while the US focus results are presented in the supplemental material (eTables 1 and 2).

Free-text responses were analyzed using an inductive qualitative approach where emergent themes were categorized and illustrative quotes extracted. The qualitative analysis was conducted by a single analyst with input from the team and was meant as a post-hoc investigation to supplement the quantitative analysis.

The study team additionally conducted a post-hoc analysis to assess non-response bias by participants’ country location between the invited and responding participants. Each survey invitee’s country was determined by extracting country names or abbreviations in the author affiliation field from the original PubMed query. If authors had multiple publications returned through the query, only the affiliation from the most recent publication was used.

All analyses were conducted in RStudio using R version 4.2.0 and the following packages: easyPubMed [6] (RRID:SCR_023570), dplyr [10] (RRID:SCR_016708), haven [11], Hmisc [12] (RRID:SCR_022497), tableone [13], and likert [14]. This study was reviewed and approved as exempt research by the Institutional Review Board (IRB) at the Boston University Medical Campus (H-44571).

## 3. Results

Our climate and health PubMed query performed on 8 March 2024 returned 20 169 matching articles from 2021–2023 and 35% of them had at least one author email address listed. After removing duplicates, we were left with 9195 authors with email addresses to whom we sent out the survey. Our survey response rate was low but comparable to other blind web-based surveys at 11.8% which corresponded to a total of 1041 useable responses from individuals who work in climate and health research or an adjacent field (see eMethods3 for response rate calculation details) [7]. We included an additional demographics module at the end of the survey after the conclusion of our scientific questions, at which point we had a drop-off rate of approximately 20%.

Responses were received from participants in at least 75 different countries, with only a quarter of responses from US-based participants (from among those who opted to report their country of residence). However, from our post-hoc analysis of author affiliations, we found that participants with a US affiliation were in fact more likely to respond to the survey compared to those affiliated with other countries; authors with US affiliations made up only 17% of invitees but 24% of respondents. China was the most common country of affiliation from the PubMed query at 19%, but China-affiliated authors were much less likely to respond to our survey, making up only 9% of the response cohort. The other most-represented countries each with approximately 4%–7% of responses include the United Kingdom, Canada, Australia, Italy, Spain, and India.

**Figure 1.**
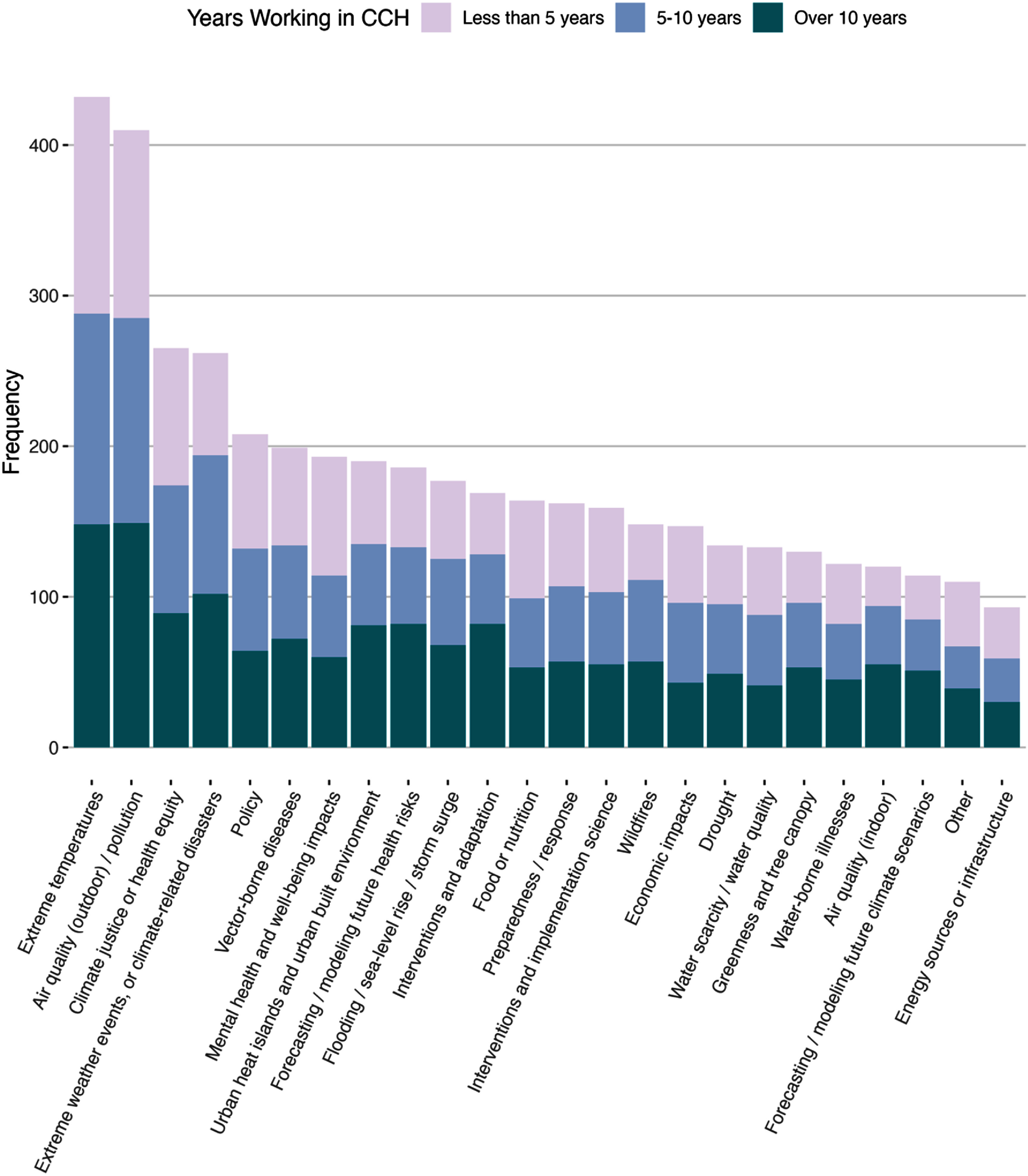
Thematic areas of climate stressors and health consequences work by frequency of report and stratified by career stage.

Table 1 shows the breakdown of respondents by U.S. focus, career duration, job sector, and demographics. A minority of survey respondents reported working primarily with US populations, making up only 16.4% of respondents (*n* = 171). There was a fairly even split between early career (*n* = 397, 38.1%), mid-career (*n* = 314, 30.2%), and established career professionals (*n* = 330, 31.7%). The vast majority of respondents worked in academia (*n* = 861, 82.7%), likely reflecting our participant search strategy that required a publication indexed in PubMed. There was also representation from government (*n* = 160, 15.4%), and healthcare (*n* = 99, 9.5%) sectors. Almost a fifth of participants declined to answer our demographic questions, which were included as a separate module at the very end of the survey. Among those who responded to the demographic questions, slightly more were male (*n* = 458, 54.5%) than female (*n* = 347, 41.3%), about 50.2% (*n* = 421) identified as White or European ancestry, and 25.9% (*n* = 217) as Asian or Asian-American. When asked about topic areas of work, there were two thematic areas that were the most common: ‘Extreme temperatures (e.g. heat, heatwaves, cold, etc)’ followed closely by ‘Air quality (outdoor)/pollution’ (figure 1). The least common topic area of work was ‘Energy sources or infrastructure (e.g. electricity consumption, fuel sources, blackouts, etc).’

**Table 1.** Survey respondent characteristics.

	Respondents *N* = 1041
Works primarily with US data	171 (16.4)

Years worked in climate and health	
Less than 5 years	397 (38.1)
5–10 years	314 (30.2)
Over 10 years	330 (31.7)

Where work [multi-select]	
Academic	861 (82.7)
Government/public sector	160 (15.4)
Healthcare	99 (9.5)
Non-governmental organization or non-profit	65 (6.2)
Industry	14 (1.3)
Other	18 (1.7)

Gender^a^	
Woman	347 (33.3)
Man	458 (44.0)
Non-binary person	2 (0.2)
Prefer not to answer	34 (3.3)
Missing	200 (19.2)

Race^a^ [multi-select]	
Asian or Asian-American	217 (20.8)
Black or African-American	44 (4.2)
Hispanic or Latino	73 (7.0)
Middle Eastern or North African	36 (3.5)
Native American, American Indian, Alaska Native, or First Nations	5 (0.5)
Pacific Islander or Native Hawaiian	2 (0.2)
White or European ancestry	421 (40.4)
Some other race	20 (1.9)
Prefer not to say	63 (6.1)
Missing	202 (19.4)

^a^ *Note*: Gender and race questions were included in a ‘Demographics’ module at the very end of the survey. Many participants chose to exit the survey after the end of the substantive questions and not fill out the demographics section. As such, this demographic information is missing for approximately one fifth of participants (*N* = 200, 19.2%). Proportions reported here are from among the total sample including missingness. Proportions reported within the text are re-calculated from among those who responded to the Demographics module.

Barriers to accessing, using, and sharing data was one of the main themes of the survey, and differences were seen by career duration of respondents (table 2). 23% of early-career respondents reported not knowing where to find climate data (*n* = 93), while this was half as common among mid-career (*n* = 36, 12%), and established career respondents (*n* = 34, 10%). However, the most common barrier reported for all groups was that ‘The data available are not in the spatial or temporal resolution that I need’ with approximately one-third of respondents endorsing this statement (*n* = 323, 31%). Slightly fewer than half of respondents reported sharing data upon request (*n* = 501, 48%), but more than half said that they would be willing to start sharing their data in the future (*n* = 571, 55%). Only 22% of respondents reported depositing their data to a data sharing platform as part of their data lifecycle strategy (*n* = 230), which was least common among early-career respondents. Among those who deposit data to a sharing platform, the most common platform selected was GitHub at 34%, with personal websites as the second most common platform at 20%. The most common barriers to sharing their own data across all career durations were ‘IRB or data use agreement (DUA) constraints’ (*n* = 295, 28%), and ‘personal preferences or constraints (e.g. no time to curate data)’ (*n* = 239, 23%). Notably, 22% of mid-career respondents also selected ‘Systemic issues in academia (e.g. tenure stress, competitiveness, etc)’ as a major barrier to sharing data (*n* = 69).

**Table 2.** Data use and sharing barriers, stratified by career duration.

	Overall *N* = 1041	Early Career *n* = 397	Mid-Career *n* = 314	Established Career *n* = 330
Barriers to acquiring, analyzing, or storing climate and health data *[multi-select]*				
I do not know where to find climate data	163 (15.7)	93 (23.4)	36 (11.5)	34 (10.3)
I do not know where to find health data	192 (18.4)	75 (18.9)	69 (22.0)	48 (14.5)
Data storage issues—data are too big	117 (11.2)	38 (9.6)	48 (15.3)	31 (9.4)
Do not have the computational power to process the data—data is too big	134 (12.9)	56 (14.1)	43 (13.7)	35 (10.6)
I do not have the software or expertise to process these types of data	198 (19.0)	87 (21.9)	61 (19.4)	50 (15.2)
The data available are not in the spatial or temporal resolution that I need	323 (31.0)	104 (26.2)	110 (35.0)	109 (33.0)
I do not experience barriers to acquiring, processing, or storing climate and health data	237 (22.8)	76 (19.1)	69 (22.0)	92 (27.9)
Other	136 (13.1)	45 (11.3)	36 (11.5)	55 (16.7)
Missing	67 (6.4)	31 (7.8)	15 (4.8)	21 (6.4)

Data end-of-life strategy *[multi-select]*				
Destroy it (as per restricted use agreement)	89 (8.5)	36 (9.1)	25 (8.0)	28 (8.5)
Archive it in house, without sharing	252 (24.2)	104 (26.2)	70 (22.3)	78 (23.6)
Archive it, sharing upon request	501 (48.1)	178 (44.8)	165 (52.5)	158 (47.9)
Deposit on a data sharing platform	230 (22.1)	67 (16.9)	80 (25.5)	83 (25.2)
Other	92 (8.8)	33 (8.3)	21 (6.7)	38 (11.5)
Missing	134 (12.9)	55 (13.9)	34 (10.8)	45 (13.6)

Would you be willing to start sharing your data in the future				
Yes	571 (54.9)	234 (58.9)	166 (52.9)	171 (51.8)
No	93 (8.9)	36 (9.1)	31 (9.9)	26 (7.9)
Missing	377 (36.2)	127 (32.0)	117 (37.3)	133 (40.3)

Barriers to sharing data *[multi-select]*				
I do not know how or where to share my data	166 (15.9)	79 (19.9)	48 (15.3)	39 (11.8)
IRB or DUA constraints prevent me from sharing the data	295 (28.3)	112 (28.2)	91 (29.0)	92 (27.9)
Personal preferences or constraints (e.g. no time to curate the data)	239 (23.0)	88 (22.2)	82 (26.1)	69 (20.9)
Worry or discomfort that data are not perfect	129 (12.4)	56 (14.1)	39 (12.4)	34 (10.3)
Cybersecurity issues (e.g. data privacy or doxxing concerns)	157 (15.1)	62 (15.6)	53 (16.9)	42 (12.7)
Lack of requirements from funders or journals	121 (11.6)	41 (10.3)	43 (13.7)	37 (11.2)
Systemic issues in academia (e.g. tenure stress, competitiveness, etc)	187 (18.0)	66 (16.6)	69 (22.0)	52 (15.8)
I do not experience barriers to sharing data	151 (14.5)	48 (12.1)	41 (13.1)	62 (18.8)
Other	89 (8.5)	37 (9.3)	18 (5.7)	34 (10.3)
Missing	128 (12.3)	53 (13.4)	33 (10.5)	42 (12.7)

When participants were asked similar questions about code (table 3), common barriers to sharing were also identified. The most common barrier to sharing code or analysis workflows was again related to personal constraints and not having time to curate code for sharing and was most frequently endorsed by mid-career respondents (*n* = 110, 35%). Worry or a concern that code was not perfect was frequently selected among early (*n* = 80, 20%) and mid-career (63, 20%), and less frequently for established career respondents (*n* = 42, 13%). Mid-career respondents were also most likely to report that they deposited their code on a code-sharing platform (*n* = 59, 19%) compared to those in early (*n* = 49, 12%) or established careers (*n* = 45, 14%). GitHub was the most popular platform where over half of depositors reported posting their code. Half of respondents reported a willingness to share code in the future (*n* = 551, 53%).

**Table 3.** Code and analysis workflow sharing barriers, stratified by career duration.

	Overall *N* = 1041	Early career *n* = 397	Mid-Career *n* = 314	Established career *n* = 330
Code end-of-life strategy *[multi-select]*				
Destroy it	30 (2.9)	15 (3.8)	6 (1.9)	9 (2.7)
Archive it in house, without sharing	303 (29.1)	124 (31.2)	82 (26.1)	97 (29.4)
Archive it, sharing upon request	458 (44.0)	170 (42.8)	153 (48.7)	135 (40.9)
Deposit on a code sharing platform	153 (14.7)	49 (12.3)	59 (18.8)	45 (13.6)
Other	90 (8.6)	33 (8.3)	23 (7.3)	34 (10.3)
Missing	151 (14.5)	59 (14.9)	38 (12.1)	54 (16.4)

Would you be willing to start sharing your code in the future				
Yes	551 (52.9)	210 (52.9)	169 (53.8)	172 (52.1)
No	155 (14.9)	61 (15.4)	43 (13.7)	51 (15.5)
Missing	335 (32.2)	126 (31.7)	102 (32.5)	107 (32.4)

Barriers to sharing code *[multi-select]*				
I do not know how or where to share my code	193 (18.5)	88 (22.2)	53 (16.9)	52 (15.8)
Personal preferences or constraints (e.g. no time to curate the code pipeline)	297 (28.5)	100 (25.2)	110 (35.0)	87 (26.4)
Worry or discomfort that code is not perfect	185 (17.8)	80 (20.2)	63 (20.1)	42 (12.7)
Cybersecurity issues (e.g. data privacy or doxxing concerns)	117 (11.2)	41 (10.3)	40 (12.7)	36 (10.9)
Lack of requirements from funders or journals	120 (11.5)	44 (11.1)	40 (12.7)	36 (10.9)
Systemic issues in academia (e.g. tenure stress, competitiveness, etc)	154 (14.8)	55 (13.9)	51 (16.2)	48 (14.5)
I do not experience barriers to sharing code	195 (18.7)	70 (17.6)	58 (18.5)	67 (20.3)
Other	90 (8.6)	35 (8.8)	19 (6.1)	36 (10.9)
Missing	126 (12.1)	52 (13.1)	33 (10.5)	41 (12.4)

**Figure 2.**
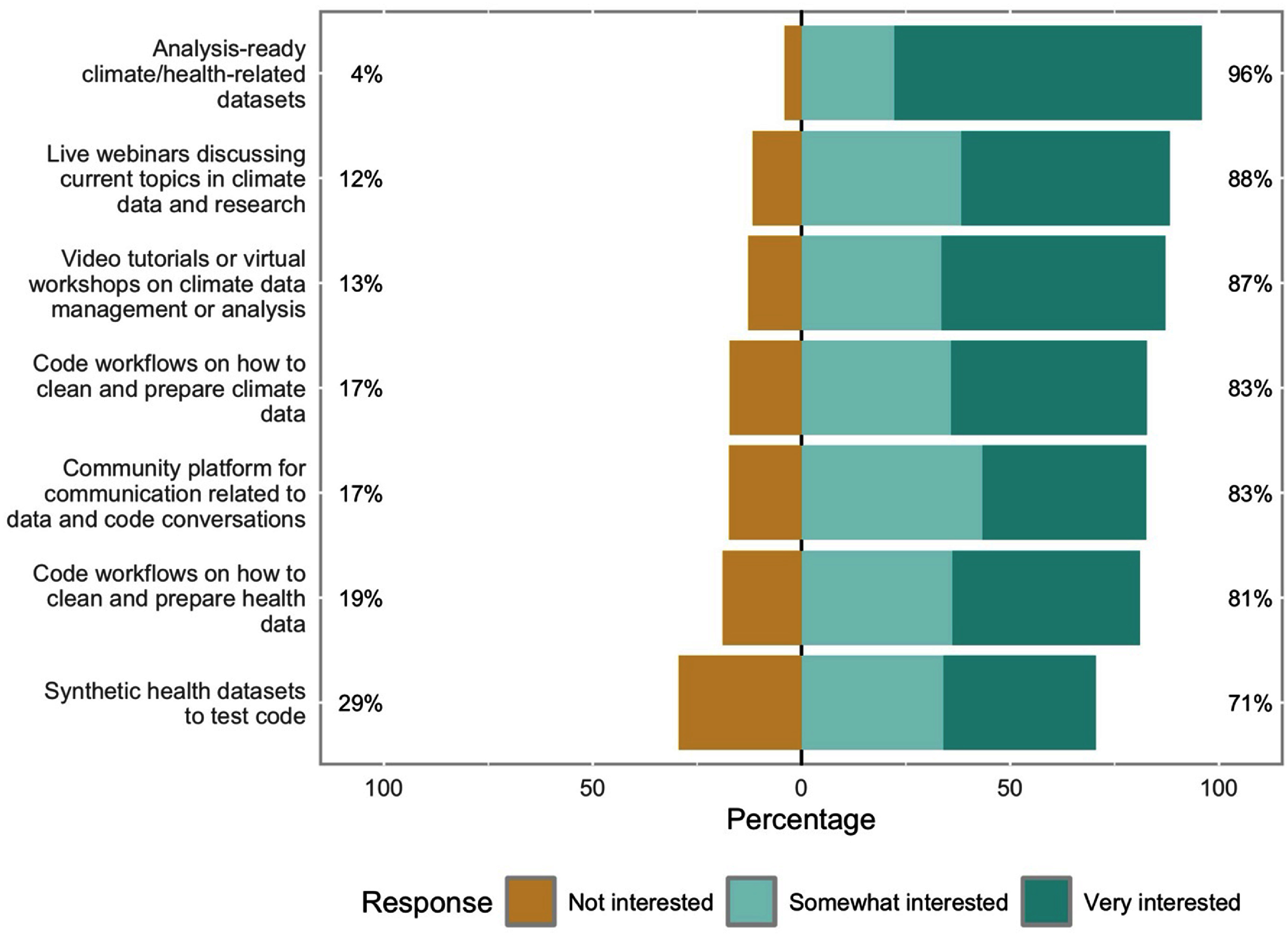
Most desired resources from climate and health survey respondents.

We asked participants what resources they most desired; all potential resources we listed were highly endorsed by respondents, but the most popular included ‘Analysis-ready climate/health-related datasets’, ‘Live webinars discussing current topics in climate data and research’, and ‘Video tutorials or virtual workshops on climate data management or analysis’ (figure 2). Among those who were not already familiar with using GitHub (*n* = 516, 50%), approximately 71% (*n* = 368) said they would like tutorials on how to use it. A smaller proportion of respondents (*n* = 342, 33%) also requested help in writing an NIH-compliant Data Management Sharing Plan.

The survey also asked participants a number of questions related to the technical details of data and software they use most frequently. Allowing respondents to select multiple selections, the most common software or coding languages used by members of this community include R (*n* = 515, 50%), ArcGIS (*n* = 290, 28%), SPSS (*n* = 290, 28%), and Python (*n* = 232, 22%). Respondents slightly preferred to use climate data that was already aggregated to administrative boundaries (*n* = 396, 49% of non-missing respondents) and preferred .csv and .xlsx file types, but a high proportion also desired data in its original gridded raster format (*n* = 334, 41% of non-missing respondents), preferably in geoTIFF and netCDF files. Those who use data primarily from the US identified census tracts, zip codes, and counties as the most useful spatial resolution for aggregated data (about 41% indicated these preferences), though at least 25% also use aggregations at the city/town, census block, and census block group levels. In the non-US data users, the city/town resolution was the most indicated at 47% of respondents with national and sub-city units approximately tied for second most desired spatial resolution for data. Allowing for multiple responses, respondents primarily use data at a daily (*n* = 417, 40%), and annual (*n* = 394, 38%) resolution, but monthly (*n* = 337, 32%), seasonal (*n* = 304, 29%), and hourly (*n* = 182, 18%) resolution data were also common. Only approximately 7% of respondents are currently using a metadata schema when curating their data (*n* = 69).

Lastly, we ended the survey with two open-ended questions and grouped responses into emergent thematic categories. In response to ‘What climate and health data would you like to use but have not been able to find?’, the most common themes included high-resolution or local data, more data on extreme weather and heat, and zoonotic and vector-borne diseases. In response to ‘Is there anything else you would like to share with us’, the most common sentiments expressed were the need for improved access to data, desire for more community/communication/collaboration, and a thank you for conducting this work.

## 4. Discussion

The findings from our survey of researchers who have published at the intersection of climate and health offer critical insights into the technical needs and current practices of this broad community of practice. While our survey response rate was low (11.8%), following a pattern of low responses found from other blind, unincentivized, web-based surveys [15], we had a total of 1041 responses from individual members of our target community across at least 75 countries and evenly representing early, mid, and established career professionals. Statisticians have proposed that survey data with such low response rates can still be reliable if the total number of responses is 500 or greater [16]. While these results may not be generalizable to the entire climate and health community, they represent an important and valuable snapshot of the opinions of those actively engaged in this work. Moreover, we conjecture that those who chose to respond to the survey may also be those most likely to have unmet data or analytic needs as well as a stronger desire to engage in critical research infrastructure activities. Our choice to identify survey participants from within PubMed may also over-represent those working in health as opposed to climate spaces, though our target population were those working at this intersection. Despite the relatively low response rate, we expect these results to capture the needs of scientists who are most interested in driving collaboration for climate and health research.

One of the most prominent findings from our survey was the consistency across career stages, as well as US and non-US data users, regarding the most common data-related challenges: the unavailability of data at the specific desired spatial or temporal scale. Early-career researchers were particularly likely to report difficulty finding climate data, highlighting an important opportunity for accessible and well-documented centralized data platforms to lower barriers to entry into the field. However, our results also suggest that simply expanding data availability alone is not enough: the next greatest challenge researchers reported was a lack of software or technical expertise to process climate/health data for their needs. This indicates a need for increased investment in technical skills education, training, and mentorship beyond just making the data more findable and useable. Finally, our finding that the majority of authors publishing about climate and health are not focused on US data underscores that future data efforts must use a global lens. Improving data access and sharing resources will allow for fruitful collaborations as well as autonomous investigations between data users and data producers, wherever they work in the world, but special attention should be given to address gaps in resource availability in low and middle-income countries to help build local capacity.

Despite widespread acknowledgment of the importance of data and code sharing, particularly in light of the 2023 data management sharing policies enacted by the NIH and other major funders as well as data sharing requirements from some publishers [17], the reuse of data and code is uncommon. A previous survey of scientists echoes our finding that insufficient time, lack of funding support, and difficulty in ensuring proper accreditation may keep researchers from sharing their data publicly [18]. However, some of the barriers to finding appropriate climate and health data could be partially addressed if data and code were shared more readily in searchable environments. Our results indicated only 22% of respondents deposit their data to a data sharing platform, and 15% respectively for their analysis code. Among those who publicly share their research data, the majority did so on sites that do not adhere to Findability, Accessibility, Interoperability, and Reusability (FAIR) [19] data principles, making it difficult for others to find and reuse. These proportions are similar to those from interviews of NSF-affiliated researchers which found that data custodians most frequently shared their data based on individual request, then by posting on a personal website, and followed distantly by depositing within a repository [20]. Development of a common environmental health sciences language may also help make published data more findable—an effort currently being undertaken by the Environmental Health Language Collaborative [21].

Changing behavior is notoriously difficult and implementation scientists highlight the need for capability, opportunity, and motivation to coincide in order to do so successfully [22]. In this case to promote open science and data reuse, addressing all three of these core principles must involve simplifying the sharing process with better data and code sharing infrastructure, additional training on FAIR principles and metadata standards, and promoting the personal benefit of depositing resources for public use. As for the first, the Generalist Repository Ecosystem Initiative from the NIH Office of Data Science Strategy is facilitating the standardization of metadata elements and centralization of data in accessible repositories such as Harvard Dataverse, Dryad, Figshare, Zenodo, and others, which will make it easier for researchers to share their prepared data for reuse [23]. The use of data sharing platforms that assign unique identifiers such as DOIs for data products will additionally provide researchers who spent considerable time and effort into creating, cleaning, and harmonizing climate and health data to be recognized and cited for this work. A lack of time was the most frequently endorsed barrier to sharing data, but notably a fifth of mid-career professionals also highlighted ‘systemic issues in academia’ as a major barrier to sharing data, similar to the aforementioned study of scientists conducted over a decade ago [18]. Both of these concerns might be partially alleviated if the important work of data curation—typically a time-consuming yet scientifically critical task—could be effectively measured, incentivized, and valued on par with other scholarly metrics of success for career advancement and promotion [24]. Researchers surveyed about data publication indicated that citation counts and download counts might both be appropriate inducements for sharing data [25]. ‘Systemic issues in academia’ may also refer to a concern about losing the ability to publish first or lack of proper accreditation, which were identified as common concerns among the surveyed NSF-affiliates as well [20]. However, there is hope that if sharing data becomes more professionally advantageous, perhaps through increased visibility, more fruitful collaborations, or enhanced recognition, researchers may be more likely to invest time and resources into doing so.

Structural barriers such as IRB or DUA constraints were also commonly endorsed and are more challenging to address. Sensitive data such as HIPAA-protected personal health data necessarily require additional data protection and security to safeguard potentially identifiable information. Increased access and sharing of derivatives of these data or resources related to their analysis would require greater participation and investment from the appropriate oversight entities. The creation of restricted access data enclaves and development of code workflows for merging public data to protected health data may be important next steps to expand the usability of sensitive data.

Many of the aforementioned barriers have been known to researchers and funders but have rarely been documented and quantified to help inform future efforts as they have been here. In fact, there has been substantial recent investment in enhancing research infrastructure and collaboration including, but not limited to, the Connecting Health Outcomes Research and Data Systems efforts within the National Institutes of Health (NIH), the Gateway Exposome Coordinating Center and the Center for Aging, Climate, & Health supported by the National Institute on Aging, the RAPID Natural Hazard and Disaster Reconnaissance Facility and interrelated Natural Hazards Engineering Research Infrastructure network both supported by the NSF, and finally this NIH-funded CAFE Research Coordinating Center. Part of CAFE’s mission is to help address research gaps to accelerate the pace of climate and health research. CAFE convenes interdisciplinary researchers across the health and physical sciences, hosts almost 1000 datasets on Dataverse with custom metadata to promote reuse and accreditation including cross-listings from NSF DesignSafe, creates and curates educational materials to help promote best practices, and aids researchers in translating their work beyond academia.

Understanding the major barriers to using and sharing climate and health data and code resources is a critical first step in addressing gaps in research. Encouragingly, the majority of our participants expressed willingness to share both data and code in the future, suggesting that with the right infrastructure and support, the climate and health community of practice may move toward more open and FAIR research standards. To this end, we urge researchers to integrate data management efforts from the outset of their projects and maintain them throughout the lifespan to simplifying the ultimate sharing process. We recommend that funders and institutions recognize the necessary work of data curation and sharing and support these activities accordingly through sustained provision of funds and supported employee time. Repositories must model aligned metadata practices and uphold DOI standards to strengthen the research data ecosystem and make data more findable. Finally, data users must accredit data creators through citation and acknowledgement to ensure researchers’ time and effort in data creation and preparation are recognized. The results of our survey suggest that if we invest in the expansion of data creation as a professional service, knowledge sharing and collaboration, and research infrastructure, that these resources will be enthusiastically adopted and help to accelerate the pace of research to practice.

## Data and code availability statement

The data that support the findings of this study are openly available at the following URL/DOI: 10.7910/DVN/3C0C7P

Supplement: CAFE Data Survey available at http://doi.org/10.1088/2752-5309/ae44c0/data1
